# Cardiovascular magnetic resonance–derived hemodynamics predict adverse outcomes after Fontan palliation

**DOI:** 10.3389/fcvm.2026.1937933

**Published:** 2026-10-21

**Authors:** Carson Platnick, Benjamin Frank, Jennifer Romanowicz, Cesar Gonzalez De Alba, Maria Chase, Amna Umer, Nita R. Chaudhuri, Jonathan Awori, Dhaval Chauhan, Christopher E. Mascio, Jai P. Udassi, Mehdi Hedjazi Moghari

**Affiliations:** 1 Department of Anesthesiology, Children's Hospital Colorado, University of Colorado School of Medicine, Aurora, CO, United States; 2 Department of Pediatrics, Section of Cardiology, Children's Hospital Colorado, University of Colorado School of Medicine, Aurora, CO, United States; 3 Department of Pediatrics, West Virginia University Medicine Children's Hospital, West Virginia University, Morgantown, WV, United States; 4 Department of Cardiovascular and Thoracic Surgery, West Virginia University Medicine Children's Hospital, West Virginia University, Morgantown, WV, United States

**Keywords:** cardiovascular magnetic resonance, flow quantification, Fontan circulation, pulmonary blood flow distribution, risk stratification

## Abstract

**Background:**

Long-term outcomes following the Fontan procedure remain heterogeneous, and robust imaging-based predictors of adverse events are limited. Patients residing at moderate-altitude may experience additional pulmonary vascular stress; however, the prognostic value of cardiovascular magnetic resonance (CMR)–derived flow parameters in this population are not well defined.

**Methods:**

We performed a retrospective cohort study of Fontan patients followed at a single moderate-altitude center who were ≥5 years post-Fontan and had undergone comprehensive CMR exams. Demographic, anatomic, and surgical data were collected. CMR-derived variables included indexed ventricular volumes and function, systemic arterial dimensions, and flow indices in the ascending and descending aorta, superior vena cava (SVC), inferior vena cava, and branch pulmonary arteries.

The primary composite outcome was death or referral/listing for heart transplantation. Time-to-event analyses from the time of CMR were performed using Kaplan–Meier estimates and Cox proportional hazards regression.

**Results:**

A total of 182 Fontan patients were included (median age at Fontan 3.2 years [interquartile range (IQR) 2.6–3.9]; median time from Fontan to CMR 10.9 years [IQR 7.7–14.2]). Over a median follow-up of 4.7 years following CMR (IQR 3.1–6.6), 14 patients (7.7%) experienced the primary outcome. In univariable Cox regression, a higher SVC flow index was significantly associated with a lower risk of the primary outcome [hazard ratio [HR] 0.70 per 0.1 L/min/m^2^ increase; 95% confidence interval [CI] 0.50–0.92; *p* = 0.008].

**Conclusions:**

In this moderate-altitude Fontan cohort, reduced CMR-derived SVC flow index was significantly associated with the primary outcome. This finding suggests that systemic venous flow merits further investigation as a prognostic marker in the Fontan population.

## Introduction

The Fontan operation represents the final palliative stage for patients born with single-ventricle congenital heart disease, creating a circulation in which systemic venous return is directed passively into the pulmonary arteries without the support of a sub-pulmonary ventricle. Since its original description, progressive refinements in surgical technique—including staged palliation, extracardiac conduits, and routine fenestration—along with advances in perioperative and longitudinal care have led to substantial improvements in early survival and long-term life expectancy for Fontan patients (1–3). As a result, the population of adolescents and adults living with Fontan physiology continues to expand, shifting clinical priorities from survival alone toward the early identification, risk stratification, and management of late Fontan-related complications (4).

Despite these advances, the Fontan circulation remains inherently unstable. The absence of a sub-pulmonary pump renders pulmonary blood flow sensitive to even modest elevations in pulmonary vascular resistance (PVR) and abnormalities in pulmonary artery (PA) compliance (5–7). Also, chronically elevated systemic venous pressure and reduced cardiac output contribute to a broad spectrum of Fontan-associated morbidities, including arrhythmias, thromboembolic events, Fontan-associated liver disease, protein-losing enteropathy, plastic bronchitis, and ultimately heart failure and the need for transplantation (2, 5, 6). Identifying early hemodynamic and structural markers that precede these adverse outcomes remains a central challenge in current Fontan care (8, 9).

Cardiovascular magnetic resonance (CMR) has emerged as the reference standard for comprehensive, noninvasive assessment and monitoring of Fontan anatomy and physiology. CMR uniquely allows for accurate quantification of ventricular volumes and function, vessel dimensions, myocardial viability, and flow measurements across both arterial and venous structures (10–12). Prior studies have demonstrated associations between CMR-derived metrics (such as ventricular dysfunction, abnormal pulmonary blood flow distribution, and reduced cardiac output), and adverse clinical outcomes in Fontan patients (13–15). However, much of the existing literature has focused on cohorts studied at or near sea level, where pulmonary vascular loading conditions may differ from those encountered at higher elevations.

Residence at moderate or high altitudes is associated with chronic hypobaric hypoxia, which leads to pulmonary vasoconstriction, increased PVR, and elevated PA pressure (16, 17). These physiologic adaptations, while often well tolerated in individuals with biventricular circulation, may pose unique challenges for patients with Fontan physiology, in whom pulmonary blood flow is entirely passive. Even small increases in PVR can significantly impair preload to the single ventricle, reduce cardiac output, and exacerbate systemic venous congestion (18, 19). Prior studies have identified elevated PVR and PA pressure as independent risk factors for Fontan failure and late morbidity (20–22).

Clinical outcome data regarding Fontan patients living at altitude remain mixed. Some reports suggest that carefully selected Fontan patients can achieve survival comparable to sea-level cohorts, particularly in the modern surgical era (23, 24). In contrast, other studies have reported higher rates of complications, reduced exercise capacity, and increased mortality among Fontan patients residing at altitude (7, 25). Importantly, most of these investigations have relied on clinical, echocardiographic, or catheterization-based assessments and have not systematically evaluated advanced CMR-derived hemodynamic and morphologic parameters as predictors of outcome.

To date, there is a lack of data examining whether CMR-based measures of vascular flow can stratify risk for adverse outcomes in Fontan patients living at altitude. This represents a knowledge gap, as such parameters may reflect the integrated physiological burden imposed by altitude-related pulmonary vascular changes on the Fontan circulation. Accordingly, the present study sought to comprehensively evaluate CMR-derived measures of hemodynamics and cardiac function in a large cohort of Fontan patients followed at a moderate-altitude tertiary referral center. Using standardized CMR techniques, we quantified pulmonary arterial flow, systemic venous return, and systemic arterial dimensions, alongside ventricular volumes and function. We then examined the associations between these parameters and a composite adverse Fontan outcome encompassing death and heart transplant referral or listing.

## Methods

### Patient selection

This retrospective, single-center cohort study included patients who underwent Fontan palliation at a tertiary care institution located at a moderate altitude (≈5,118 ft) between 1999 and 2021 and who subsequently underwent CMR evaluation following the Fontan procedure. The study protocol was approved by the local Institutional Review Board, with a waiver of informed consent due to the retrospective nature of the analysis. Patients were eligible for inclusion if they had undergone at least, one clinically indicated CMR examination performed ≥5 years after Fontan completion, and if the corresponding CMR report and clinical data were available in the medical record. The ≥5-year post-Fontan threshold was selected because it corresponds to the time point at which routine CMR surveillance of Fontan physiology is commonly initiated at our center. A total of 182 unique patients met the inclusion criteria. One post-Fontan CMR study per patient was included in the final analysis, specifically the last CMR study obtained prior to the occurrence of the outcome or censoring in patients with multiple studies.

### Demographic data and clinical data

Demographic, anatomic, and surgical characteristics were abstracted from the electronic medical record. Demographic variables included sex, age at Fontan completion, primary cardiac diagnosis, body surface area (BSA) at the time of CMR acquisition, and residential home elevation. Home elevation was determined using the patient's primary residence zip code and expressed in feet above sea level. Clinical timing variables included the interval from Fontan completion to CMR acquisition, as well as the time from CMR acquisition to the first composite adverse event or censoring. Primary cardiac diagnoses were categorized, and anatomic characteristics were defined according to dominant ventricular morphology. Surgical variables included Fontan type, and whether a Damus–Kaye–Stansel (DKS) anastomosis had been performed.

Clinical outcome data were also obtained through detailed review of the medical record. The primary outcome was a composite adverse Fontan outcome, defined *a priori* as the occurrence of death, and heart transplant referral or listing. Patients were considered to have met the primary outcome at the time of the first qualifying event. Secondary outcomes included established Fontan-associated morbidities including Fontan-associated liver disease, protein-losing enteropathy, plastic bronchitis, and stroke. Time-to-event variables were calculated from the date of CMR acquisition to the first occurrence of any component of the primary outcome.

### CMR imaging protocol and data

CMR examinations were performed using 1.5-T or 3.0-T magnetic resonance imaging scanners (Philips Healthcare, Best, the Netherlands). Initially, two-dimensional cine steady-state free precession sequences were acquired to assess cardiac anatomy and ventricular function. Subsequently, two-dimensional phase-contrast (PC) gradient-echo sequences were used to quantify arterial and venous blood flow. Magnetic resonance angiography was performed to evaluate extracardiac vascular anatomy.

Ventricular volumes and function, including end-diastolic volume (EDV), end-systolic volume (ESV), and ejection fraction (EF), were abstracted from the clinical CMR reports. Net flow volumes were recorded for the ascending aorta (AAO), right PA (RPA), left PA (LPA), superior vena cava (SVC), and inferior vena cava (IVC). Systemic-to-pulmonary collateral flow was calculated as AAO flow minus the sum of SVC and IVC flows. The ratio of SVC to AAO flow was also derived as a measure of relative systemic venous return. Ventricular volumes and vascular flows were indexed to BSA at the time of CMR.

All flow and volumetric measurements were performed by pediatric cardiologists and radiologists experienced in clinical pediatric CMR interpretation. Well before this study, at the inception of our CMR program, our center conducted a formal inter- and intra-observer reproducibility analysis of CMR-derived ventricular volumes and flow quantification. This analysis informed a standardized institutional protocol for vessel contouring and flow analysis, ensuring consistency across observers and studies in our clinical practice.

For comparative analyses, patients were stratified according to the presence or absence of the primary outcome. Patients who experienced secondary outcomes without meeting criteria for the primary outcome were included in the no primary outcome group.

### Statistical analysis

All statistical analyses were conducted using JMP statistical software, JMP Statistical Discovery LLC, JMP®, ver. 18, Cary, NC, USA, 2024. Continuous variables are reported as median with interquartile range [IQR], and categorical variables are reported as counts with percentages. Continuous variables were compared using the Wilcoxon rank-sum test, and categorical variables using the chi-square or Fisher's exact test, as appropriate. All tests were two-sided, with a *p*-value <0.05 considered statistically significant.

To derive a threshold value for stratification of event-free survival, a receiver operating characteristic (ROC) curve was constructed with the primary composite outcome (death or heart transplant referral/listing) as the classification variable. The area under the curve (AUC) with its 95% confidence interval (CI) was calculated to quantify discriminative performance. The optimal cut-point was identified using the Youden index (J = sensitivity + specificity−1), and the sensitivity and specificity at this threshold were reported. This data-derived threshold was then applied to dichotomize the cohort. Freedom from the primary outcome was estimated using the Kaplan–Meier method, with between-group comparisons performed using the log-rank test; patients without a composite event were censored at the date of last known follow-up. Harrell's concordance index (C-index) was also calculated.

Cox proportional hazards regression was performed to evaluate associations between CMR-derived cardiac function and hemodynamic parameters and the primary outcome. Hazard ratios (HRs) and corresponding 95% CIs were calculated. Overall model fit and statistical significance were assessed using likelihood ratio tests.

Given the limited number of composite events, we pre-specified a parsimonious multivariable Cox model restricted to two variables to test independence rather than to develop a prediction model; no automated or stepwise variable selection was performed. Collinearity was assessed using pairwise correlation coefficients and variance inflation factors. To evaluate the stability of the HR estimates, nonparametric bootstrapping was performed with 2,500 resamples. Bootstrap results were summarized as the median and IQR of the resampled HRs. The proportional hazards assumption was assessed using Schoenfeld residuals, with a non-significant correlation between residuals and time considered consistent with proportionality.

Not all CMR-derived parameters were available for every patient, as measurements were abstracted from clinical reports and individual sequences were occasionally not acquired or not reported. Analyses were performed on a complete-case basis, with each analysis restricted to patients who had the relevant variable(s) available; no data was imputed. The number of patients contributing to each analysis is reported in the corresponding tables and figures.

## Results

### Study population characteristics

A total of 182 patients with Fontan circulation were included in the study. The cohort was predominantly male (53%), with a median age at Fontan completion of 3.2 years [IQR 2.6–3.9]. At the time of CMR acquisition, the median BSA was 1.43 m^2^ [IQR 1.16–1.69], and patients resided at a median home elevation of 5,221 ft [IQR 4,793–5,518], consistent with moderate-altitude exposure (26).

The median interval from Fontan completion to CMR was 10.9 years [IQR 7.7–14.2], with a median time from CMR to event or censoring of 4.7 years [IQR 3.1–6.6]. Hypoplastic left heart syndrome (35%) and tricuspid atresia (30%) were the most common underlying diagnoses. Left- and right-dominant ventricular morphologies were similarly represented (53% vs. 47%). The majority of patients underwent extracardiac conduit Fontan palliation (95%). A DKS anastomosis had been performed in 42% of patients. Baseline demographic, anatomic, and surgical characteristics are summarized in Table 1.

**Table 1:** Baseline demographic, anatomic, and surgical characteristics of fontan patients residing at a moderate-altitude center.

Characteristics of the Study Population (*N* = 182)	
Demographic	
Gender (Male)	96 (53%)
Age at Fontan completion, years	3.2 [2.6, 3.9]
Body surface area at CMR, m^2^	1.43 [1.16, 1.69]
Home elevation, feet	5,221 [4,793, 5,518]
Time from Fontan to CMR acquisition, years	10.9 [7.7, 14.2]
Time from CMR to event or censoring, years	4.7 [3.1, 6.6]
Diagnosis	
Hypoplastic left heart syndrome	64 (35%)
Tricuspid atresia	54 (30%)
Pulmonary atresia	15 (8%)
Double inlet or double outlet ventricle	22 (12%)
Unbalanced atrioventricular canal	12 (7%)
Other single-ventricle diagnoses	15 (8%)
Anatomic Characteristics	
Dominant left ventricle	97 (53%)
Dominant right ventricle	85 (47%)
DKS anastomosis	76 (42%)
Fontan Surgical Characteristics	
Extracardiac conduit Fontan	173 (95%)

Continuous variables are presented as median [interquartile range], and categorical variables as number (percentage). CMR: Cardiovascular Magnetic Resonance; DKS: Damus-Kaye-Stansel.

### Clinical outcomes

During follow-up, 14 patients (7.7%) experienced the primary outcome (Table 2). Components of the primary outcome included death in 2 patients (1.1%) and heart transplant referral or listing in 12 patients (6.6%). Fontan-associated morbidities were common. Fontan-associated liver disease was identified in 47 patients (25.8%), protein-losing enteropathy in 16 patients (8.8%), plastic bronchitis in 11 patients (6.0%), arrhythmia in 12 patients (6.6%), and stroke in 3 patients (1.6%). Among patients meeting the primary outcome, the median time from CMR examination to first adverse event was 1.2 years [IQR 0.5–2.5].

**Table 2:** Clinical outcomes and fontan-related complications.

Post-Fontan Complications in the Overall Cohort (103/182)	
Primary Outcome Components	
Death	2 (1.1%)
Heart transplant referral/listing	12 (6.6%)
Secondary Outcomes	
Fontan-associated liver disease	47 (25.8%)
Protein-losing enteropathy	16 (8.8%)
Plastic bronchitis	11 (6.0%)
Sustained arrhythmia	12 (6.6%)
Stroke	3 (1.6%)
Composite Adverse Fontan Outcome (Primary Endpoint)	
Any component of the primary outcome	14 (7.7%)
Time from CMR to event, years	1.2 [0.5, 2.5]

Composite adverse Fontan outcome was defined as death, heart transplant referral, or listing. Data are presented as number (percentage) or median [interquartile range].

### CMR parameters

CMR-derived measures of flow distribution stratified by primary outcome are summarized in Table 3. Patients who experienced a primary adverse event had a significantly lower SVC flow index than those without events [0.54 [IQR 0.46–0.84] vs. 0.82 [0.66–1.07] L/min/m^2^, *p* = 0.011]. On ROC analysis, the SVC flow index discriminated patients who met the primary outcome with an AUC of 0.74 (95% CI 0.56–0.92); the Youden-optimal threshold was 0.66 L/min/m^2^, yielding a sensitivity of 70% and a specificity of 75% (Youden index 0.45). Also, SVC flow index showed fair discrimination for complications, with a C-index of 0.73 (95% CI: 0.55–0.90). The SVC to AAO flow ratio was likewise significantly lower in patients who experienced the primary outcome [0.22 [IQR 0.18–0.29] vs. 0.30 [0.24–0.36], *p* = 0.020]. In contrast, AAO, RPA, LPA, and IVC flow indices did not differ significantly between groups (all *p* > 0.05), and systemic-to-pulmonary collateral flow was comparable (*p* = 0.935).

**Table 3:** Cardiovascular Magnetic resonance–derived measures of hemodynamics stratified by primary outcome.

Variable	Association of Cardiac Function, Vessel Size and Hemodynamics with Primary Outcome				
	No-Primary Outcome median [IQR]	N	Primary Outcome median [IQR]	N	*p*-value
Systemic Flow					
AAO flow index (L/min/m^2^)	2.90 [2.43, 3.40]	152	2.65 [2.33, 3.28]	10	0.432
Pulmonary Blood Flow Distribution					
RPA flow index (L/min/m^2^)	1.19 [0.95, 1.50]	133	1.09 [0.74, 1.36]	8	0.281
LPA flow index (L/min/m^2^)	1.01 [0.83, 1.20]	135	0.80 [0.49, 1.07]	8	0.084
Systemic Venous Return					
SVC flow index (L/min/m^2^)	0.82 [0.66, 1.07]	143	0.54 [0.46, 0.84]	10	**0**.**011**
IVC flow index (L/min/m^2^)	1.53 [1.22, 1.87]	147	1.53 [1.33, 1.92]	10	0.888
Systemic-to-Pulmonary Collateral					
AAO-IVC-SVC (L/min/m^2^)	0.41 [0.28, 0.74]	123	0.46 [0.24, 0.97]	9	0.935
Relative Systemic Venous Return					
SVC flow/AAO flow	0.30 [0.24, 0.36]	141	0.22 [0.18, 0.29]	10	**0**.**020**

AAO: ascending aorta; RPA: right pulmonary artery; LPA: left pulmonary artery; SVC: superior vena cava; IVC: inferior vena cava.

*P* values reflect between-group comparisons.

Table 4 presents the association between ventricular size, EF, and the primary outcome stratified by ventricular dominance. A higher proportion of patients with a dominant RV experienced the primary outcome compared with those with a dominant LV, although this difference did not reach statistical significance (*p* = 0.054). Among patients with dominant LV morphology, indexed EDV, indexed ESV, and EF did not differ significantly between those with and without the primary outcome. However, this finding should be interpreted with caution, given the small number of LV-dominant patients (*N* = 4) who experienced the primary outcome. In contrast, significant differences were observed in the RV-dominant subgroup, where patients with the primary outcome exhibited substantially higher indexed ventricular volumes and lower EF (*p* < 0.01).

**Table 4:** Cardiovascular magnetic resonance–derived measures of ventricular size and function stratified by primary outcome.

Association of Cardiac Function with Primary Outcome			
Variable	No-Primary Outcome	Primary Outcome	*p*-value
LV Dominant	*N* = 92	*N* = 4	
EDV index (mL/m^2^)	85.48 [74.69, 104.81]	82.00 [64.74, 93.79]	0.436
ESV index (mL/m^2^)	42.15 [32.76, 53.37]	32.05 [28.03, 42.93]	0.140
EF (%)	50.94 [46.51, 57.20]	55.44 [51.29, 64.02]	0.172
RV Dominant	*N* = 74	*N* = 10	
EDV index (mL/m^2^)	103.15 [87.75, 119.47]	189.02 [105.39, 233.79]	**0**.**009**
ESV index (mL/m^2^)	55.98 [44.82, 68.85]	126.28 [62.16, 180.11]	**0**.**003**
EF (%)	46.21 [38.81, 51.69]	33.69 [22.80, 39.51]	**0**.**003**

Comparison of indexed end-diastolic volume (EDV), indexed end-systolic volume (ESV), and ejection fraction (EF) between patients with and without the primary outcome, stratified by systemic ventricular dominance [left ventricular (LV) vs. right ventricular (RV)]. Continuous variables are presented as median [interquartile range].

*P* values reflect between-group comparisons within each ventricular dominance subgroup.

### Event-free survival analysis

Following CMR, patients were stratified by SVC flow index using the Youden-derived optimal cutoff of 0.66 L/min/m^2^. Kaplan–Meier analysis (Figure 1) demonstrated early and sustained separation of survival curves, with significantly reduced event-free survival in patients with lower SVC flow index (*χ*^2^ = 6.35, df = 1, *p* = 0.012).

**Figure 1:**
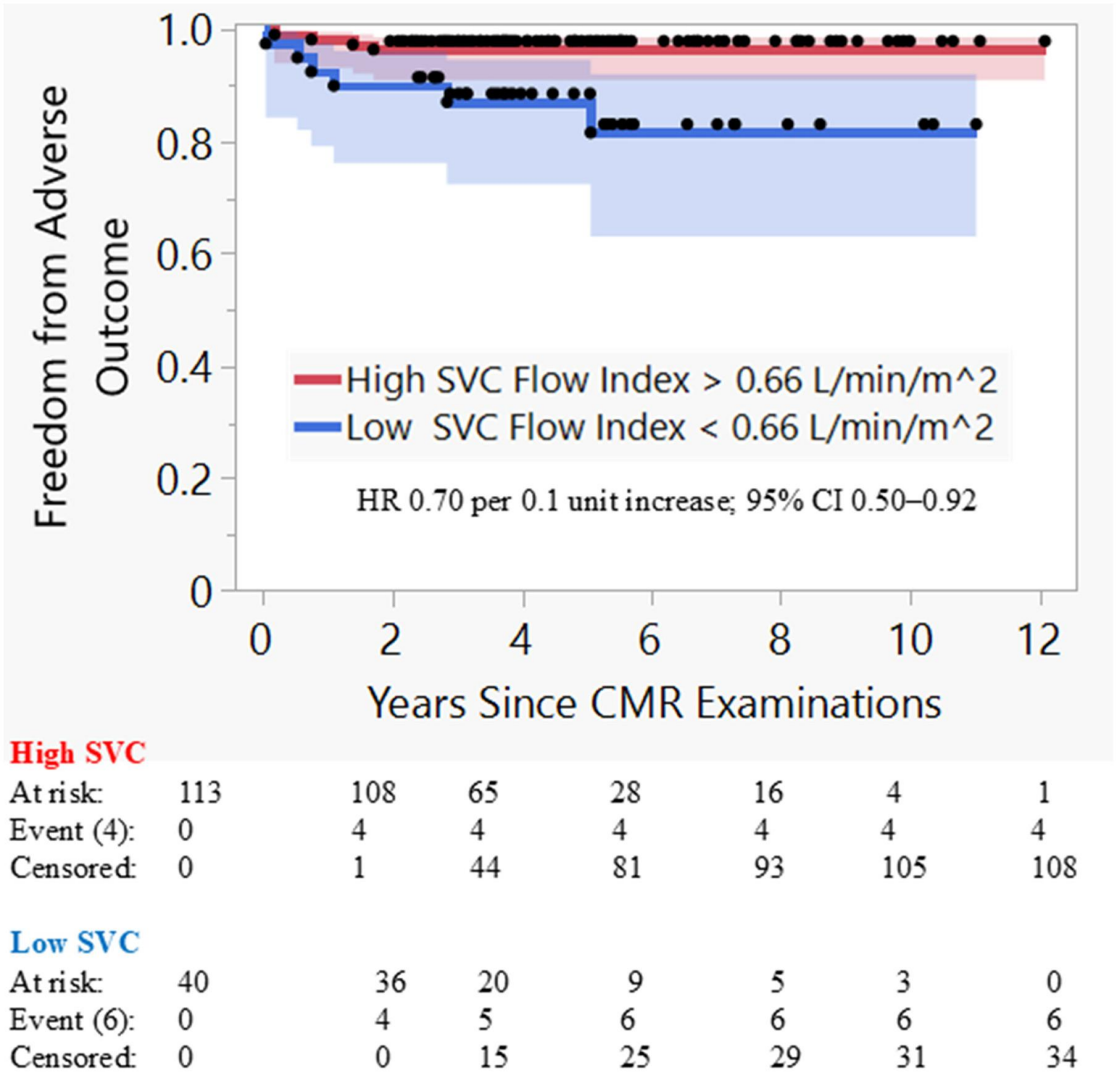
Kaplan–meier analysis of event-free survival stratified by superior vena cava (SVC) flow index. Kaplan–Meier curves demonstrating event-free survival following CMR, stratified by SVC flow index using a cutoff of 0.66 L/min/m^2^. Patients with lower SVC flow index (<0.66 L/min/m^2^) in blue exhibited significantly reduced event-free survival compared with those with higher SVC flow (≥0.66 L/min/m^2^) in red. The difference between the two groups was statistically significant (log-rank *p* = 0.012).

Event-free survival also differed significantly according to ventricular morphology (Figure 2). Patients with RV morphology experienced a more rapid decline in event-free survival compared with those with LV morphology, with early and progressive separation of the survival curves (*χ*^2^ = 4.32, df = 1, *p* = 0.038).

**Figure 2:**
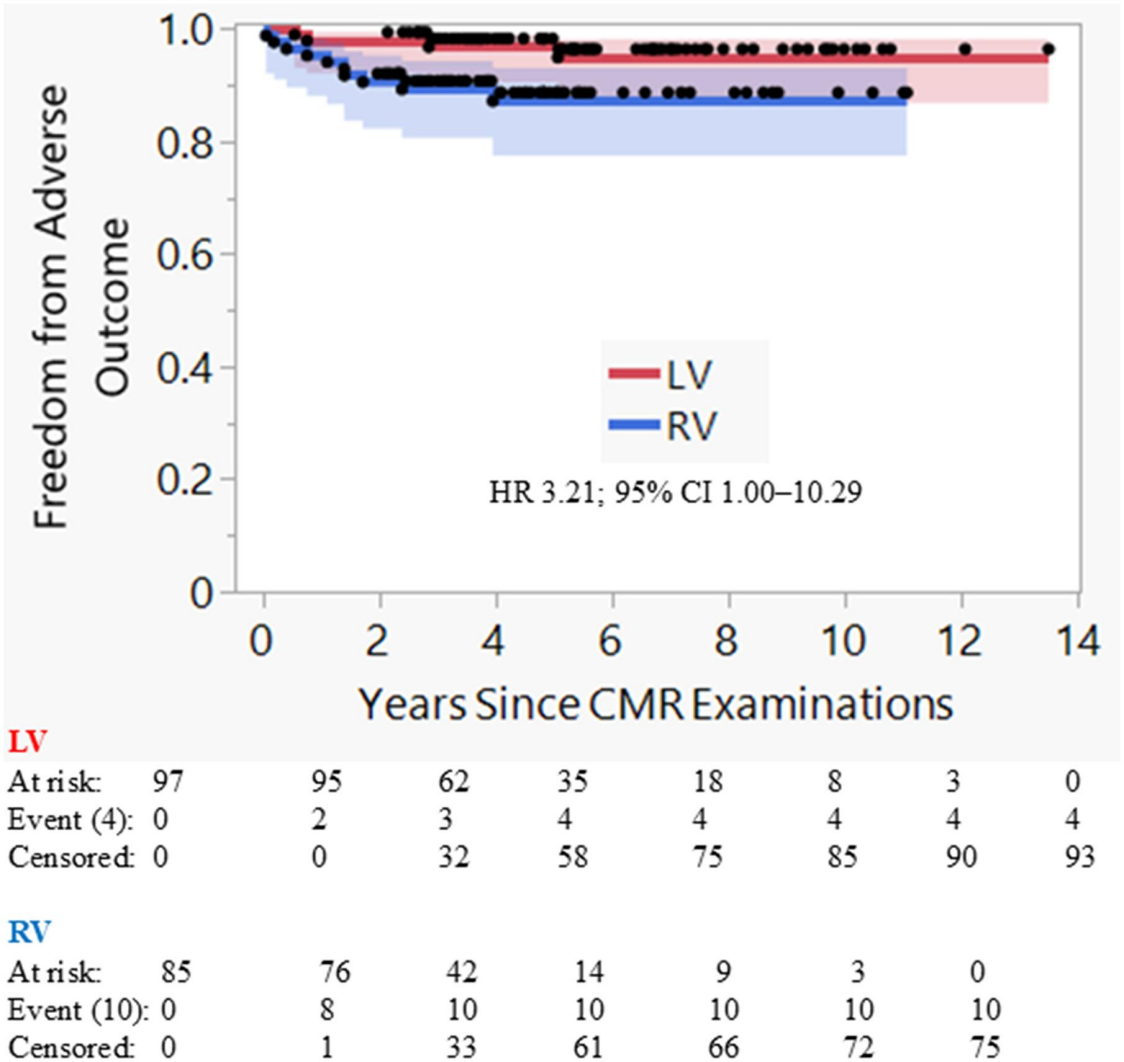
Kaplan–meier analysis of event-free survival stratified by ventricular morphology. Kaplan–Meier curves demonstrating event-free survival following CMR, stratified by ventricular morphology. Patients with RV morphology exhibited significantly reduced event-free survival compared with those with LV morphology (log-rank *p* = 0.038).

### Univariate cox proportional hazards analysis

In univariable Cox regression, a higher SVC flow index was independently associated with a lower hazard of the primary outcome, with each 0.1 L/min/m^2^ increase conferring a 30% reduction in hazard (HR 0.70; 95% CI 0.50–0.92; *p* = 0.008). Ventricular morphology also influenced risk: dominant RV morphology was associated with a higher hazard than dominant LV morphology (HR 3.21; 95% CI 1.00–10.29; *p* = 0.049). Sex and age at Fontan completion were not significantly associated with the primary outcome.

### Multivariable cox proportional hazards analysis

To evaluate whether the association between SVC flow index and adverse outcome was independent of EF, a multivariable Cox proportional hazards model including SVC flow index and EF was constructed. Despite the limited number of events, both variables remained significantly associated with the outcome. The overall model demonstrated strong predictive value (*χ*^2^ = 14.59, df = 2, *p* < 0.001).

After adjustment for EF, a higher SVC flow index remained independently associated with a lower risk of adverse outcome, with each 0.1 L/min/m^2^ increase conferring an approximately 28% reduction in hazard (adjusted HR 0.72; 95% CI 0.53–0.93; *p* = 0.008). Higher EF was similarly associated with reduced risk (adjusted HR 0.92 per 1% increase; 95% CI 0.86–0.97; *p* = 0.006). Bootstrap resampling supported the stability of both associations. For SVC flow index, the bootstrap median HR per 0.1 L/min/m^2^ increase was 0.71 [IQR 0.64–0.77], closely approximating the original estimate of 0.72. For EF, the bootstrap median HR was 0.92 [IQR 0.89–0.94], essentially identical to the original estimate. Both distributions remained entirely below 1.0 across the IQR, consistent with a persistent protective association. Collinearity between SVC flow index and EF was negligible (Spearman |*ρ*| < 0.1, R^2^ = 0.008, variance inflation facto*r* = 1.01), confirming that the two variables provided largely independent prognostic information. The proportional hazards assumption was not violated for either SVC flow index (*p* = 0.60) or EF (*p* = 0.63).

## Discussion

In this moderate-altitude Fontan cohort, we demonstrated that CMR-derived measures of vascular flow distribution, specifically SVC flow index, and ventricular morphology are independently associated with the primary outcome. These findings extend the prognostic utility of CMR beyond conventional volumetric assessment and provide mechanistic insight into the hemodynamic phenotype associated with adverse events in this cohort; whether moderate altitude contributes to this phenotype cannot be determined from the present data.

### SVC flow index as a hemodynamic marker

Our results align with a growing body of literature emphasizing the importance of regional flow dynamics over global ventricular function alone. Rathod et al. demonstrated that maldistribution of pulmonary blood flow assessed by CMR was associated with reduced exercise capacity in Fontan patients, highlighting the physiologic importance of efficient pulmonary flow pathways independent of EF or cardiac output (15). Similarly, Rijnberg et al. showed that flow imbalance and increased viscous energy loss within the total cavopulmonary connection are associated with inefficient Fontan hemodynamics and adverse clinical trajectories (27).

Within this framework, the association between lower SVC flow index and the primary outcome represents a novel and clinically meaningful observation. The Fontan circulation is uniquely preload-dependent and exquisitely sensitive to PVR. In a moderate-altitude environment, even modest elevations in PVR, driven by chronic hypoxic pulmonary vasoconstriction, may attenuate effective cavopulmonary flow, a hypothesis that requires validation in multicenter studies, ideally incorporating a sea-level comparison cohort. Because PVR was not directly measured in this study, the mechanism underlying reduced SVC flow index cannot be established. A lower SVC flow index may reflect elevated PVR or impaired venous return efficiency, but this interpretation remains hypothetical. If confirmed, CMR-based flow quantification could offer a complementary adjunct for estimating pulmonary vascular load (28).

Several alternative non–mutually exclusive mechanisms merit consideration. First, a lower indexed SVC flow may be influenced by anatomical differences in SVC caliber, which were not systematically assessed in this study. Second, systemic venovenous collateral formation diverting blood from the SVC to the atrium, thereby bypassing the pulmonary vascular bed, may contribute to reduced measured SVC flow while simultaneously lowering effective pulmonary blood flow and promoting systemic desaturation. Third, differences in preload and in single-ventricle filling may alter the proportion of systemic venous return traversing the SVC. These mechanisms may coexist, and the present data cannot distinguish their relative contributions. Accordingly, the association between reduced SVC flow index and adverse outcomes should be regarded as a marker of an unfavorable hemodynamic state rather than direct evidence of any single underlying mechanism.

### Ventricular morphology and outcomes

Ventricular morphology further modified risk in our cohort. Patients with a dominant RV morphology had significantly worse event-free survival compared with those with a dominant LV morphology. This finding is consistent with prior studies demonstrating inferior long-term outcomes in patients with a systemic RV (29), likely attributable to inherent differences in myocardial wall thickness, ventricular geometry, fiber architecture, and susceptibility to progressive systolic dysfunction under chronic systemic loading conditions. The convergence of an unfavorable ventricular substrate with the hemodynamic challenges imposed by moderate altitude may further accelerate adverse remodeling in this subgroup.

### Contributions to literature

This study contributes to existing literature in two important respects. First, it characterizes a relatively large Fontan cohort residing at moderate altitude, a population that remains underrepresented in contemporary outcome studies. Second, it leverages advanced CMR-based flow quantification to evaluate SVC flow index as a prognostic marker, an approach not previously reported in long-term Fontan outcome analyses. While prior investigations have examined pulmonary flow distribution and energy loss within the Fontan pathway, SVC-specific indexed flow has not, to our knowledge, been systematically evaluated or identified as a significant predictor of adverse events.

### Limitations

Several limitations warrant acknowledgment. First, the retrospective, single-center design introduces the potential for selection bias. As a single-center cohort residing at a uniform moderate elevation, our design lacks a sea-level comparator and does not permit assessment of altitude as an effect modifier. The role of altitude in the observed associations is therefore hypothesized on physiological grounds rather than demonstrated and requires validation in multicenter studies spanning a range of elevations. CMR examinations were performed as part of routine clinical surveillance rather than at standardized, protocol-defined intervals; consequently, variability in the timing of imaging relative to clinical status may have influenced the observed associations.

Many clinically relevant variables known to influence Fontan outcomes were not available for or incorporated into our analysis, including New York Heart Association functional class, cardiopulmonary exercise capacity, resting oxygen saturation, natriuretic peptide levels, catheterization-derived hemodynamics including Fontan (central venous) pressure and pulmonary vascular resistance, pulmonary vasodilator therapy, arrhythmia burden, and severity of Fontan-associated liver disease. Any of these factors could confound the observed association between SVC flow index and adverse outcomes.

Most importantly, only 14 patients experienced the composite outcome. Even our deliberately parsimonious two-variable model therefore approaches the event-to-variable ratio lower than the conventional minimum of 10 events per variable. This raises the possibility of model overfitting and coefficient instability and is directly reflected in the wide confidence intervals surrounding the SVC flow index hazard ratios. Although we mitigated this by pre-specifying rather than selecting covariates, avoiding collinear predictors, and applying bootstrap internal validation, the multivariable estimates should be regarded as exploratory. The magnitude of the reported HRs should be interpreted with caution, and external validation in larger, multicenter cohorts is required before these associations can inform clinical risk stratification.

In addition, detailed histories of prior PA interventions and concomitant medications with potential hemodynamic effects, including pulmonary vasodilator therapy, were not systematically captured and may have influenced vascular dimensions or flow metrics. We analyzed the last available CMR prior to the event or censoring rather than the first examination or a protocol-standardized surveillance study. This approach was chosen because most patients underwent only a single post-Fontan CMR, which precluded time-dependent covariate modeling. However, selecting the examination closest to the outcome may introduce reverse-causation bias if patients approaching clinical deterioration were imaged nearer to the event, when hemodynamics may already have been abnormal. The observed association between reduced SVC flow index and adverse outcomes should be interpreted with this potential bias in mind, and prospective studies using standardized surveillance imaging at pre-defined intervals will be needed to confirm the temporal relationship.

Finally, the majority of patients at our center underwent fenestrated extracardiac conduit Fontan palliation, reflecting contemporary institutional practice; generalizability to non-fenestrated or alternative Fontan configurations may therefore be limited.

### Future directions

Prospective, multicenter studies comparing these findings with sea-level cohorts are necessary to validate the observed associations and refine altitude-specific risk stratification models. Integration of advanced imaging techniques such as four-dimensional (4D) flow CMR (30) may further elucidate three-dimensional flow patterns, quantify energy loss within the Fontan pathway, and enhance physiologic risk assessment. Correlating CMR-derived hemodynamic signatures with exercise physiology, biomarkers of end-organ dysfunction, and pulmonary vascular imaging could deepen mechanistic understanding and inform individualized management strategies. Finally, investigation of modifiable therapeutic targets including pulmonary vasodilator therapy and catheter-based or surgical interventions aimed at optimizing SVC-directed pulmonary blood flow, represents a compelling avenue for future translational research informed by the present findings.

## Conclusion

In this single-center cohort of patients with Fontan circulation residing at moderate altitude, CMR-derived SVC flow index provided independent prognostic information beyond conventional volumetric parameters. Specifically, a lower indexed SVC flow was associated with an increased risk of the primary outcome, suggesting that comprehensive CMR-based hemodynamic assessment may represent a candidate marker for risk stratification in the Fontan population that requires prospective, multicenter validation before clinical application. Dominant RV morphology was an additional independent predictor of adverse events.

## Data Availability

The raw data supporting the conclusions of this article will be made available by the corresponding author upon reasonable request and with permission from the institution.
